# Post-Stroke Brain Health Monitoring and Optimization: A Narrative Review

**DOI:** 10.3390/jcm12237413

**Published:** 2023-11-29

**Authors:** Eric L. Stulberg, Perminder S. Sachdev, Anne M. Murray, Steven C. Cramer, Farzaneh A. Sorond, Kamakshi Lakshminarayan, Behnam Sabayan

**Affiliations:** 1 Department of Neurology, University of Utah, Salt Lake City, UT 84112, USA; eric.stulberg@hsc.utah.edu; 2 Centre for Healthy Brain Ageing (CHeBA), University of New South Wales, Sydney, NSW 2052, Australia; p.sachdev@unsw.edu.au; 3 Neuropsychiatric Institute, Prince of Wales Hospital, Sydney, NSW 2031, Australia; 4 Berman Center for Outcomes and Clinical Research, Minneapolis, MN 55415, USA; amurray@bermancenter.org; 5 Department of Medicine, Geriatrics Division, Hennepin Healthcare Research Institute, Minneapolis, MN 55404, USA; 6 Department of Neurology, University of California Los Angeles, Los Angeles, CA 90095, USA; sccramer@mednet.ucla.edu; 7 California Rehabilitation Institute, Los Angeles, CA 90067, USA; 8 Department of Neurology, Northwestern University Feinberg School of Medicine, Chicago, IL 60611, USA; farzaneh.sorond1@northwestern.edu; 9 Division of Epidemiology and Community Health, School of Public Health, University of Minnesota, Minneapolis, MN 55455, USA; laksh004@umn.edu; 10 Department of Neurology, Hennepin Healthcare Research Institute, Minneapolis, MN 55404, USA

**Keywords:** stroke, brain health, cognitive impairment, recovery, rehabilitation

## Abstract

Significant advancements have been made in recent years in the acute treatment and secondary prevention of stroke. However, a large proportion of stroke survivors will go on to have enduring physical, cognitive, and psychological disabilities from suboptimal post-stroke brain health. Impaired brain health following stroke thus warrants increased attention from clinicians and researchers alike. In this narrative review based on an open timeframe search of the PubMed, Scopus, and Web of Science databases, we define post-stroke brain health and appraise the body of research focused on modifiable vascular, lifestyle, and psychosocial factors for optimizing post-stroke brain health. In addition, we make clinical recommendations for the monitoring and management of post-stroke brain health at major post-stroke transition points centered on four key intertwined domains: cognition, psychosocial health, physical functioning, and global vascular health. Finally, we discuss potential future work in the field of post-stroke brain health, including the use of remote monitoring and interventions, neuromodulation, multi-morbidity interventions, enriched environments, and the need to address inequities in post-stroke brain health. As post-stroke brain health is a relatively new, rapidly evolving, and broad clinical and research field, this narrative review aims to identify and summarize the evidence base to help clinicians and researchers tailor their own approach to integrating post-stroke brain health into their practices.

## 1. Introduction

Cerebrovascular disease leads to approximately 116 million disability life years (DALYs) lost, representing the second leading cause of disability globally [1,2]. As acute stroke care and primary prevention have improved substantially over time in the US, age-standardized death rates from stroke have significantly decreased [3]. However, there is an increasing number of community-dwelling stroke survivors. The estimated prevalence of stroke survivors in the US has increased from about five million individuals in 2002 to more than seven million in 2018 [4]. By 2030, an estimated USD 56 billion per year will be lost in the US due to reduced productivity attributed to stroke [5].

Currently, post-acute stroke neurological care is mainly focused on secondary prevention to reduce the risk of a recurrent event. While rigorous secondary prevention is crucial to mitigate the risk of a subsequent stroke, optimizing brain health more broadly can vastly improve post-stroke physical, cognitive, and social functioning. In the following sections, we define post-stroke brain health and a clinical approach to monitoring it. We then discuss approaches to broadly optimize post-stroke brain health. Lastly, we discuss future directions of the field, including the use of telerehabilitation, neuromodulation, enriched environments, interventions focused on multi-morbidity, and improving equity with respect to optimal post-stroke brain health.

The primary focus of this review is on brain health after an ischemic stroke since the vast majority of strokes are ischemic; however, many of these concepts also apply to brain health after a hemorrhagic stroke or global cerebral ischemic event such as cardiac arrest. We hope that clinicians caring for survivors of stroke come away confident in their understanding of the current practices for monitoring and optimizing post-stroke brain health, comfortable with adapting the latest evidence to create a clinical paradigm tailored to their specific patient populations, and understanding what research advances lie on the clinical-translation horizon. Additionally, we hope researchers and policymakers alike are inspired to develop new post-stroke brain health innovations at the cellular, individual, and population levels.

## 2. Aims, Materials, Methods of Search Strategy, and Limitations

The main objective of this narrative review is to provide clinicians and researchers alike with a broad overview of the current state of the art in post-stroke brain health medicine. We a priori determined what categories of post-stroke brain health would be most relevant to review. Given the large breadth of research and clinical guidelines specifically relating to etiology-specific secondary stroke prevention, post-stroke rehabilitation methods, spasticity management, and transitions of care, we excluded these topics to limit the scope of this review to topics not as well covered elsewhere. However, these topics are essential for post-stroke brain health, and we urge clinicians to familiarize themselves with these topics as well.

A search of articles related to the topic of this paper was conducted via the use of PubMed, Scopus, and Web of Science databases up to October 2023. Identification terms were chosen to reflect our a priori categories: post-stroke brain health, post-stroke recovery, post-stroke guidelines, post-stroke monitoring, post-stroke physical activity, vascular cognitive impairment, post-stroke cognitive impairment, post-stroke physical activity, post-stroke nutrition, stroke and (each vascular category), neurovasculome, neurovascular unit, stroke and (each psychosocial category), stroke and (each lifestyle category), and updates in stroke recovery. Articles cited by articles produced by our search terms or articles that cited articles produced by our search terms were also used. Additional articles were included at the request of reviewers as well as at the discretion of co-authors. No restrictions regarding the year of the publication were placed. Only articles with English versions (either as original language or translation) were used.

Limitations of this review include its narrative nature. We as an authorship group do not represent a consensus panel. However, this review does provide one of the first summaries of the current state of the field and gives actionable clinical and research recommendations from an authorship group representing diverse expertise. Additionally, we are transparent with the level of evidence used to derive our recommendations and provide a large source of references for additional reading.

## 3. Defining and Framing Post-Stroke Brain Health

Brain health can be defined as (1) the maintenance of neurologic function across all forms of pathophysiology by preventing accumulation of brain injury, (2) the promotion of repair following brain injury, and (3) the honing of compensatory mechanisms from non-injured areas of the brain [6,7,8]. Unlike treatments initiated in response to a diagnosed brain disease, brain health management takes a proactive approach by empowering individuals, physicians, and public health systems to take steps to mitigate the often-insidious accrual of damage over time. Optimizing brain health includes risk factor modification to improve long-term outcomes, engaging in activities that enhance the brain’s plasticity and advantageous remodeling of the neurovascular unit, and growing the brain’s connectivity as a form of resilience against pathologic insults [6,9,10]. Applying a brain health framework, the narrower term “post-stroke brain health” can be defined as the state of preserved brain functional connectivity and structural integrity after stroke; the brain’s intrinsic ability to heal and repair via angiogenesis, neurogenesis, and cell-to-cell crosstalk in the ischemic penumbra; and the potential for functional compensation when there is irreversible damage. Similar stroke burdens can lead to different functional outcomes [11,12,13]. Determinants of post-stroke brain health include the burden of damage to elegant brain structures and critical functional networks [14,15,16,17,18,19,20]; the degree of atrophy of total brain volume; and the integrity of key cellular, vascular, and metabolic functions [21,22].

Optimizing post-stroke brain health aims to achieve the best possible physical, cognitive, and social functions for a given stroke burden. Mechanisms to achieve favorable post-stroke brain health include: (1) minimizing any additional damage to key structures or networks; (2) preventing neurodegenerative changes such as parenchymal loss and breakdown of the neurovascular unit; and (3) supporting neuroplasticity to counteract the effects of diaschisis, repair damage, and promote increased activity in compensatory neural pathways [23,24]. While out of the scope of this review to cover the underlying basic science of each of these goals, the translation of these goals to clinical research and practice is paramount to post-stroke brain health. Secondary stroke prevention involves minimizing additional damage. Ameliorating post-stroke cognitive decline and enhancing the integrity and function of the brain–endothelium connection encompasses the prevention of neurodegeneration. Counteracting diaschisis—the malfunction of a cerebral site distant from the stroke due to metabolic derangements, neurovascular uncoupling, and deafferentation from axonal damage—relies on individual rehabilitation and personalized treatments.

## 4. Monitoring Post-Stroke Brain Health and Neuroprognostication

With increasing attention to post-stroke brain health, a detailed understanding of patient outcomes becomes imperative. This underscores the need to move away from non-specific global outcome measures such as the modified Rankin scale and Barthel index, which provide crude, low granularity measures of post-stroke functional outcomes. Assessment of domain-specific outcome measures following stroke is needed to both study and clinically monitor the multiple individual facets of post-stroke brain health [25,26]. More granular outcome measures should be thoughtfully considered and a priori included in future stroke research and clinical trials. A recent review article lays out a framework for future outcome measure selection and implementation that we hope becomes more widespread, thereby accelerating advances in optimizing post-stroke brain health with targeted, specific interventions and measures [27].

**Figure 1:**
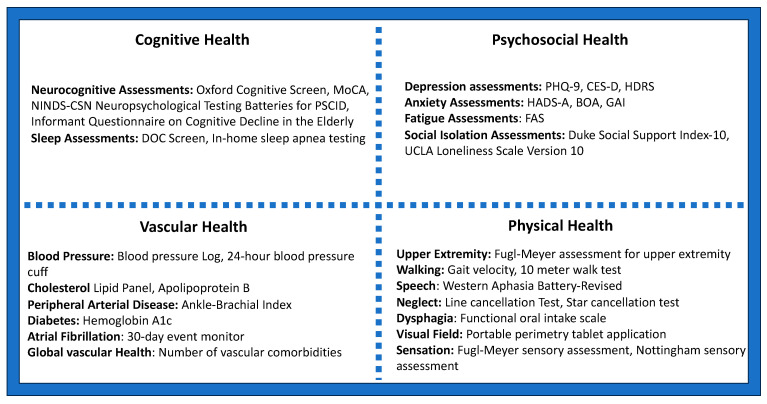
Domains of post-stroke brain health and examples of tools for domain-specific monitoring [25,27,28,32,33,34,35,36]. Key: MoCA = Montreal Cognitive Assessment; NINDS-CSN = National Institute of Neurological Disorders and Stroke—Canadian Stroke Network; PSCID = post-stroke cognitive impairment and dementia; DOC = depression, obstructive sleep apnea, cognition; PHQ-9 = Patient Health Questionnaire-9; CES-D = Center for Epidemiologic Studies Depression Scale; HDRS = Hamilton Depression Rating Scale; Hospital Anxiety and Depression Scale—Anxiety; BOA = Behavioral Outcomes of Anxiety Scale; GAI = Geriatric Anxiety Inventory; FAS = Fatigue Assessment Scale.

Building off this prior work, we propose that clinicians and researchers alike consider four distinct yet intertwined domains of post-stroke brain health: cognition, psychosocial health, physical functioning, and global vascular health. Figure 1 displays various potential metrics that could be used to monitor and evaluate post-stroke brain health in clinical practice or in future research studies. We recommend that aspects of each domain be assessed by clinicians shortly following medical stabilization of stroke (i.e., at time of discharge from the inpatient stroke unit), at all additional transition points (e.g., at time of transition from inpatient rehabilitation facility or skilled nursing facility to home), during the subacute period (e.g., at a clinic visit 3 months following stroke), and then every 6–12 months thereafter in the chronic phase of stroke. These may be monitored by a neurologist, a primary care clinician, physiatrist and rehabilitation team, or some combination depending on setting [28]. By monitoring these domain-specific measures of post-ischemic brain health over time, clinicians can provide more personalized treatment. Additionally, survivors of stroke and their families and caregivers can be educated on what to monitor within each domain based off clinical assessments at each time point mentioned above.

While Figure 1 covers multiple aspects of post-stroke brain health, it nonetheless represents just one possible framework, derived from a variety of studies, guidelines, and the opinions of the author group. As few studies or paradigms have comprehensively attempted to address post-stroke brain health, Figure 1 represents just one possible framework. We encourage future studies to validate clinical approaches to comprehensively measuring and monitoring the multi-faceted aspects of post-stroke brain health.

In addition to the domain-based testing examples presented in Figure 1, biomarker-based neuroprognostication is also advancing rapidly and will also likely impact the field in the near future. Studies have shown that increased white matter hyperintensity volume on MRI is associated with worse post-stroke cognitive outcomes, and younger volumetric brain age is associated with better sensorimotor and functional outcomes [21,29]. EEG is also under study for prognostication following stroke. Resting state congruent beta frequency in the primary motor cortex on the side of the stroke relative to the rest of the cortex may be associated with improved motor outcomes when measured 3 weeks following stroke [30]. This extends to testing during perioperative periods, such as carotid revascularization, where multi-modal intraoperative monitoring may help predict and improve outcomes [31].

## 5. Approach to Optimizing Management of Post-Stroke Brain Health

**Figure 2:**
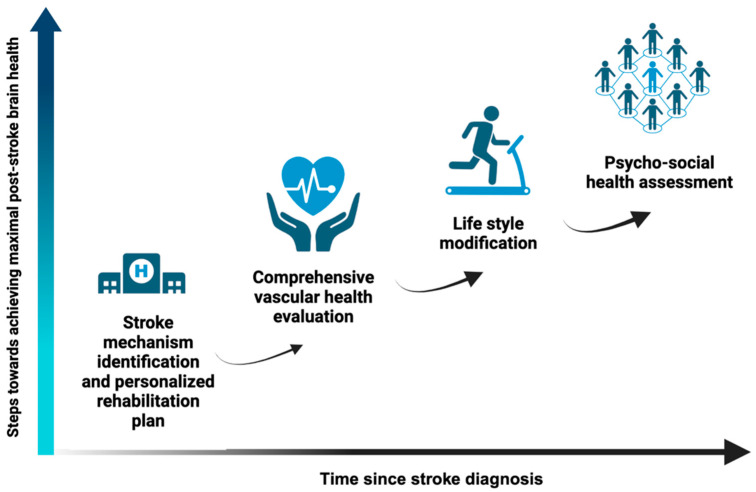
Stepwise approach to achieve and maintain post-stroke brain health.

Trajectories of cognitive and physical function over the lifetime of a stroke survivor are varied, with determinants ranging from molecular processes to healthcare delivery models. This complexity requires a multi-faceted approach to optimize and improve post-stroke brain health. We review the current evidence and propose actions that patients and clinicians can take using a stepwise approach, starting with precision-based acute and subacute stroke and rehabilitation management and advancing to comprehensive vascular health, lifestyle modifications, and psychosocial risk factors (Figure 2, created using BioRender.com under agreement number KX2658GZTJ). While precision-based care and population health seem at first glance contradictory, we believe they go hand-in-hand in improving post-stroke brain health. Individually tailored secondary stroke management and physical–neuropsychological rehabilitation will help each individual maximize their post-stroke brain health, while population-based interventions will improve post-stroke vascular health and promote adoption of lifestyle changes and psychological well-being.

### 5.1. Identification of Stroke Mechanism, Etiology-Specific Secondary Management, and Personalized Rehabilitation

The immediate goals following a new ischemic stroke include reperfusion as appropriate; prevention of medical complications post-stroke; application of secondary prevention measures; and initiation of physical, cognitive, and social rehabilitation. Up to 15% of survivors of a first-time ischemic stroke will develop a second stroke within a year [37]. Recurrent stroke is strongly associated with greater cognitive decline and functional impairment [38,39]. Identifying the specific etiology for a patient’s stroke is paramount to enable cause-specific secondary prevention. However, about 10–40% of all ischemic strokes are presumed cryptogenic [40]. As diagnostic techniques in stroke care advance, we foresee improvement in identifying stroke mechanisms and secondary prevention moving further towards a precision-based and individualized approach [41,42]. Additionally, as artificial intelligence, advanced imaging techniques, and various post-stroke biomarkers develop, we anticipate an increasing ability to better predict how specific rehabilitation interventions can be individualized to each survivor of stroke as well as to better tailor clinical trial inclusion/exclusion criteria to deal with the heterogenous causes and outcomes of stroke recovery [43].

### 5.2. Addressing Comprehensive Vascular Health

While etiology-specific management is essential, improvement of overall vascular health is also important for functional and physical recovery, preservation of cognition, and secondary prevention. For example, studies suggest that cardiac rehabilitation and intensive cardiovascular risk management may lead to greater functional improvement and better motor recovery in survivors of stroke [44,45,46]. In the following paragraphs, we summarize the relation of different vascular health components with post-stroke brain health. Table 1 displays clinical considerations and areas for future research 

#### 5.2.1. Blood Pressure

Hypertension occurs in up to 75% of survivors of stroke, frequently uncontrolled, with 40% having long term hypertension [47]. A recent meta-analysis found that the greater the degree of lowering blood pressure after stroke, the greater the risk reduction in all-cause cardiovascular events and recurrent stroke [48]. The evidence on blood pressure control mitigating the risk of post-stroke cognitive decline and dementia is mixed. The Perindopril Protection Against Recurrent Stroke Study (PROGRESS) trial showed that antihypertensive therapy is associated with lower risk of cognitive decline and dementia, especially in those with recurrent stroke [49]. However, subsequent clinical trials did not replicate this finding, though they had key limitations including short duration of follow up and young age of participants [50,51]. Further, acute blood pressure lowering immediately following stroke was not associated with improved post-stroke depression in one clinical trial, though there is scant literature on chronic outpatient blood pressure levels and post-stroke depression [52]. As such, more studies are needed to evaluate and determine how different long-term blood pressure goals following stroke affect a range of outcomes, including mood, cognition, and physical function [32]. The intended and unintended effects of different blood pressure goals are unlikely to follow a “one-size fits all” pattern, and future studies should consider how stroke etiology and other health characteristics impact the role of blood pressure in post-stroke brain health. It may be that the change in blood pressure from pre-stroke levels to post-stroke levels is more important than the absolute value of specific blood pressure goals. As such, studies are needed on the influence of change in outpatient blood pressure and blood pressure variability following stroke with cognitive, physical, and recovery outcomes. In the meantime, we recommend a blood pressure goal of <130/80 in line with the AHA/ASA’s blood pressure guidelines if there are no contraindications such as frailty, increased fall risk, severe symptomatic intracranial stenosis, or other contraindications or side effects [35,53].

#### 5.2.2. Diabetes and Glycemic Impairment

Impaired glycemic control is a common risk factor for stroke and subsequent outcomes. A meta-analysis estimated that about 33% (95% confidence interval: 28–38%) of patients with ischemic stroke have diabetes mellitus (DM) [54]. Higher hemoglobin A1C at the time of stroke is associated with higher rates of recurrent stroke, worse functional outcomes, and higher rates of mortality [55,56]. Post-stroke DM is associated with post-stroke cognitive decline and dementia [38,57]. A recent study suggests a potential non-linear relationship between hemoglobin A1C and post-stroke cognitive impairment [58]. However, it remains unclear if improvement in hemoglobin A1C following stroke independently improves post-stroke cognitive and functional outcomes and what, if any, is the ideal target hemoglobin A1C to mitigate the risk of post-stroke dementia. The American Heart Association/American Stroke Association (AHA/ASA) guidelines recommend using metformin with the addition of either a glucagon-like peptide-1 receptor agonist (GLP1) or sodium-glucose cotransporter 2 inhibitor (SGLT2) in patients with type 2 diabetes following stroke depending on other comorbid conditions, irrespective of hemoglobin A1C [35]. GLP1s are theorized to reduce the risk of cognitive decline risk through a variety of mechanisms [59]. The ongoing semaglutide cardiovascular outcomes trial (SOUL) randomized clinical trial includes cognitive secondary endpoints that will provide key insights into the efficacy of GLP1 medications in reducing cognitive decline in patients with DM and established cardiovascular diseases including stroke [60].

#### 5.2.3. Hyperlipidemia

Hyperlipidemia is common in survivors of stroke, with an estimated 27% of survivors of myocardial infarction and/or stroke having persistent dyslipidemia as measured by total cholesterol ≥ 200 mg/dL [61]. There are a variety of guideline recommendations for its management. The range of recommendations following stroke includes use of statins and other cholesterol-lowering medications to achieve LDL goals <70 mg/dL based on the AHA/ASA recommendations to as low as LDL <40 mg/dL based off the European Society of Cardiology/European Atherosclerosis Society Dyslipidemia Guidelines in certain patients with multiple cardiovascular events such as recurrent stroke within 2 years [62,63]. Additionally, early statin use may provide a neuroprotective role and improve functional outcomes above and beyond their role as secondary prevention agents [64]. With respect to post-stroke cognitive impairment, a small exploratory pilot trial called Prevention of Decline in Cognition after Stroke Trial (PODCAST) found that intensive cholesterol lowering (LDL-cholesterol <1.3 millimole/liter (~50 mg/dL) vs. LDL-cholesterol <3.0 mmol/L (~116 mg/dL)) was associated with better post-stroke attention and executive cognition as well as functional status through a 30-month follow-up period, although this trial did not meet its primary endpoint of a significant difference in the Addenbrooke’s Cognitive Examination-Revised score [65]. Moreover, only 28.6% of participants in the intensive cholesterol lowering arm met their LDL target, and the trial failed to meet its mean difference goal in LDL levels between study arms. More research is needed to determine if certain patients with stroke—such as those with intracranial atherosclerosis or severe small vessel disease—can benefit cognitively from intensive control of blood cholesterol levels. Additionally, there is observational research that the ratio of HDL/LDL may have prognostic value in cardiovascular and cerebrovascular health; research is needed to determine how this ratio affects a variety of post-stroke brain health domains independent of individual LDL and HDL levels [66].

#### 5.2.4. Heart Failure and Atrial Cardiopathy

It is estimated that about 11–18% of patients with stroke suffer from symptomatic heart failure, with an even higher proportion demonstrating evidence of left ventricular dysfunction [67]. In addition to an elevated rate of recurrent strokes, patients with heart failure have higher rates of covert infarcts found on MRI, which are associated with cognitive decline [68,69,70]. Additionally, heart failure with either preserved or reduced ejection fraction is associated with higher risk of dementia [71]. Atrial cardiopathy may also be associated with dementia, independent of stroke or atrial fibrillation [72]. Possible mechanisms of cognitive impairment in heart failure and structural heart dysfunction include cerebral hypoperfusion, subclinical chronic cardioembolic disease, and increased deposition of amyloid plaque in the brain from an increase in natriuretic peptides [73,74]. A pharmaco-epidemiological study provided evidence that angiotensin-converting enzyme inhibitors may improve cognition in patients with heart failure, independent of their effect on blood pressure [75]. Two trials have evaluated whether angiotensin receptor–neprilysin inhibitors (ARNIs) are associated with better cognitive function relative to an angiotensin-converting enzyme (ACE) inhibitor or angiotensin receptor blocker in participants with heart failure. Neither trial found a difference in their primary cognitive outcomes, though one did find less brain amyloid in the ARNI trial arm (URL: https://www.clinicaltrials.gov (accessed on 10 August 2023); unique identifier: NCT02884206) [76]. Similar studies examining medication effects on cognitive trajectory are needed that also examine patients with the dual diagnosis of stroke and heart failure. The Atrial Cardiopathy and Antithrombotic Drugs in Prevention After Cryptogenic Stroke—Cognition and Silent Infarcts (ARCADIA-CSI) trial, a sub-study of ARCADIA (URL: https://www.clinicaltrials.gov (accessed on 10 August 2023); unique identifier: NCT03192215), will evaluate whether anti-platelet medications differ from anticoagulation medications in their effect on silent infarct burden and cognition in stroke survivors with atrial cardiopathy. There is some evidence that the use of guideline-directed medical therapy with the lowest tolerable blood pressure leads to an absolute risk reduction in morbidity and mortality in patients with heart failure; however, it is unclear which blood pressure goals would be most advantageous for patients with comorbid history of stroke and heart failure on outcomes such as cognition, mood, motor recovery, and cardiopulmonary endurance [77].

#### 5.2.5. Peripheral Arterial Disease and Carotid Disease

Peripheral arterial disease (PAD) is associated with higher risk of ischemic stroke and is a frequent comorbidity in patients with stroke, with one study finding that about a third of survivors of stroke may have subclinical PAD [78,79,80,81]. There is emerging evidence that “polyvascular disease”—defined as greater than ≥50–70% stenosis or symptomatic stenosis in multiple vascular beds such as limb, coronary, or cerebral—places patients at particularly high risk for ischemic stroke and may benefit from more specific management to modify risk of cardiovascular events [78,79,80]. Additionally, PAD is known to be an independent risk factor for impaired physical functioning and has been associated with worse functional recovery in patients with stroke [82,83]. As such, further research is needed to determine if recovery after stroke in patients with comorbid PAD can be improved with a more aggressive therapeutic regimen (e.g., addition of cilostazol, more aggressive lipid management than standard of care, combination anti-thrombotic therapy, or revascularization in symptomatic limbs). Atherosclerosis of the internal carotid artery is highly studied in relation to secondary stroke prevention, with growing interest in the efficacy of transcarotid artery revascularization in symptomatic carotid disease as well as the role of nonstenotic carotid disease in presumed cryptogenic strokes [84,85]. While it is out of the scope of this review to delve into the latest research and advances in the management of carotid disease from a secondary prevention perspective, carotid disease is also associated with post-stroke cognitive impairment [86]. In the currently enrolling Carotid Revascularization and Medical Management for Asymptomatic Carotid Stenosis 2 (CREST-2) randomized clinical trial, a sub-study called CREST-Hemodynamics (CREST-H) will evaluate which, if any, carotid revascularization technique can improve cognitive outcomes in patients with asymptomatic stenosis of the internal carotid artery as compared to medical management [87]. While survivors of stroke are excluded from CREST-H, the findings may spur research into how management of carotid disease affects post-stroke cognition.

#### 5.2.6. Atrial Fibrillation

Atrial fibrillation (Afib) is likely the cause of ≥15% of strokes and is associated with increased risk of post-stroke cognitive decline [88,89,90,91,92]. One observational study showed oral anticoagulation reduced the risk of dementia in patients with Afib, relative to those not on anticoagulants [93]. While the primary analytic sample was comprised of both individuals with and without a history of stroke, there was statistically significant effect modification suggesting a stronger association in those with a history of ischemic stroke [93]. A meta-analysis of nine studies found that direct oral anticoagulation may reduce the risk of dementia in Afib more so than Warfarin, though the authors urge caution against overinterpretation of their findings [94]. Key questions on the effects of Afib on post-stroke brain health persist, including the cognitive impact of different modalities of rhythm control or atrial appendage occlusion and how the burden of Afib (proportion of time someone is in Afib) affects cognition [95,96]. Most studies on the impact of Afib on cognition are among individuals without stroke; more studies are needed to specifically define the role of Afib in post-stroke physical and cognitive functioning and whether various treatment options have differential impacts on post-stroke functional status and brain health.

#### 5.2.7. Chronic Kidney Disease

Chronic kidney disease (CKD) occurs in roughly 20–35% of patients with ischemic stroke and is increasingly recognized as a risk factor for stroke via its effects on chronic inflammation, accelerated atherosclerosis, and uremia, among other mechanisms [97,98]. CKD has been associated with disability, higher risk of institutionalization, and worse cognitive impairment following stroke [97,99,100,101]. Subclinical cerebral ischemia, particularly in small vessel territories, is theorized to drive at least some of the increased risk [97,102]. While no guidelines exist on the management of co-occurring CKD and stroke, current practice recommendations include aggressive blood pressure management with a goal <120/80, aggressive lipid management in non-dialysis-dependent patients, treatment with an SGLT-2 inhibitor in patients with diabetic CKD and an estimated glomerular filtration rate >30, and a low threshold for screening for Afib [97].

**Table 1:** Recommended clinical considerations and areas for future research in vascular determinants of post-stroke brain health.

Target Area	Clinical Considerations	Level of Evidence	Areas for Future Research
*Intracranial vascular integrity*	Consider cilostazol and/or isosorbide mononitrate in lacunar stroke survivors to improve cognitive and functional outcomes [103].	Phase II clinical trial	Develop additional medication regimens to maximize post-stroke neurovasculome function.
*Global vascular health*	Consider referring survivors of stroke for cardiac rehabilitation assessment and personalized interventions in addition to standard post-stroke rehabilitation to improve functional and motor recovery [44,45].	Observational research	Develop pragmatic trials and real-world studies assessing multi-morbidity interventions.
*Blood pressure*	Aim for strict long-term outpatient blood pressure control in patients with stroke and hypertension, taking into account the degree of extra- and intracranial atherosclerosis [35,48,104].	Secondary prevention guidelines, meta-analysis of clinical trials	Determine optimal long-term outpatient blood pressure goals with respect to post-stroke cognition and mood and if specific goals have differential effects based on various patient and stroke characteristics such as age, location of stroke, etiology of stroke, and comorbidities.
	Aim for a long-term, outpatient blood pressure goal of <130/80 if no side effects or other contraindications such as frailty and fall risk [35,48,104].		Determine the clinical significance of change in outpatient blood pressure following stroke as well as chronic outpatient blood pressure variability in post-stroke cognitive impairment, post-stroke physical recovery, and recurrent cardiovascular/cerebrovascular events.
			Determine if certain blood pressure medication classes have neuroprotective effects independent of their effects on blood pressure reduction.
*Diabetes*	Consider an SGLT-2 inhibitor for those with heart failure or chronic kidney disease or a GLP-1 agonist for secondary stroke and cardiovascular prevention in stroke patients with diabetes in addition to metformin if no contraindications [35].	Secondary prevention guidelines	Determine if there is a protective effect of GLP-1 agonists on post-stroke cognitive impairment.
	Aim for a long-term HbA1C of ≤7% in most patients [35].		Determine the optimal hemoglobin A1C target for prevention of post-stroke cognitive impairment.
*Lipids*	In patients with strokes caused by intracranial or extracranial atherosclerosis, or with a comorbid atherosclerotic condition such as coronary artery disease, more intensive LDL target of <40–55 and/or reduction in LDL by >50% can be considered for optimal secondary prevention [63,105].	Secondary prevention guidelines, post hoc analysis of clinical trial	Determine how change in different hyperlipidemia markers like low-density lipoprotein, HDL/LDL ratio, and various apolipoproteins impacts post-stroke cognition and mood and if this impact varies by different stroke and patient characteristics.
			Determine if certain medication classes such as statins or psck9-inhibtors have neuroprotective effects independent of their effects on hyperlipidemia biomarkers.
*Heart Failure*	Ensure guideline-directed medical therapy for heart failure is instituted, particularly that patients are on angiotensin-converting enzyme (ACE) inhibitors, angiotensin II receptor blockers, or angiotensin receptor neprilysin inhibitor if no contraindication given possible cognitive protection [75,106].	Observational research, heart failure guidelines	Determine if anticoagulation improves cognitive trajectories following strokes in patients with heart failure with varying ejection fractions without atrial fibrillation.
	Consider early identification and management of comorbid atrial fibrillation in patients with heart failure.		
*Peripheral Arterial Disease and Carotid Disease*	Consider screening for peripheral arterial disease with an ankle–brachial index in patients with functional limitations not explained by the stroke itself and/or in patients with significant intracranial or extracranial atherosclerosis [78,82].	Observational research, post hoc analysis of clinical trial	Determine the utility of early stenting, bypass, angioplasty, aggressive lipid management, and various anti-thrombotic regimens in improving functional outcomes in patients with comorbid peripheral arterial disease and stroke.
	If evidence of peripheral arterial disease, consider aggressive lipid management, anti-platelet management, and/or referral to peripheral vascular specialist.		Determine which, if any, patients with comorbid carotid disease and stroke would benefit cognitively from carotid revascularization.
*Atrial Fibrillation*	Early recognition of atrial fibrillation and timely initiation of anticoagulation in patients with stroke determined to be cardioembolic may lead to improved cognitive outcomes [88,89,93].	Observational research	Determine what, if any, burden of atrial fibrillation (measured as time or percentage of time in atrial fibrillation) among patients with paroxysmal atrial fibrillation predisposes to cognitive decline following stroke.
			Determine what, if any, treatment options for atrial fibrillation improve post-stroke cognitive trajectories including rate control, rhythm control, atrial appendage occlusion, and anticoagulation.
			Determine the role of anticoagulation on cognitive outcomes in patients with embolic source of undetermined significance and/or atrial cardiopathy.
*Chronic Kidney Disease*	Aim for a blood pressure goal of <120/80 in patients with comorbid chronic kidney and stroke if there are no contraindications [97].	Secondary prevention guidelines, expert consensus statement.	Determine if chronic kidney disease independently predisposes to atrial fibrillation and if prolonged monitoring for atrial fibrillation can impact post-stroke brain health following stroke.
	Consider the addition of an SGLT-2 inhibitor in patients with diabetic CKD and comorbid stroke to prevent further progression of CKD [35,97].		Determine how chronic uremic toxins and CKD treatments impact vascular function and intracranial hemodynamics as well as neuroinflammation, blood–brain barrier breakdown, and oxidative-stress-related neuronal damage.
			Determine what, if any, treatment options following stroke can mitigate silent cerebral small vessel ischemia following stroke in patients with comorbid chronic kidney disease.

### 5.3. Lifestyle Modification

In addition to vascular medical management, lifestyle modifications following stroke are a key component to optimizing post-stroke brain health. While often cited as a justification for dual anti-platelet therapy for secondary prevention in the setting of intracranial atherosclerosis disease, the Stenting and Aggressive Medical Management for Preventing Recurrent stroke in Intracranial Stenosis (SAMMPRIS) clinical trial also included a rigorous lifestyle modification program focused on nutrition, physical activity, and smoking cessation [107]. A post hoc analysis of SAMMPRIS found that greater physical activity had the largest independent association with mitigating ischemic stroke recurrence, highlighting the importance of lifestyle modifications for secondary prevention [108]. Table 2 displays clinical considerations and areas for future research.

#### 5.3.1. Physical Activity

Physical activity is a key factor to improving multiple aspects of post-ischemic stroke brain health. A 2020 Cochrane review concluded that cardiopulmonary aerobic exercise with or without resistance exercise may help reduce post-stroke disability, particularly with respect to improved mobility and endurance [109]. Both resistance training and aerobic cardiopulmonary training can also improve post-stroke balance [109]. A recent post hoc analysis of the *Efficacy of Fluoxetine—a Randomised Controlled Trial in Stroke* (EFFECTS) clinical trial found that survivors of stroke who were more physically active over time had improved physical functioning and less disability at 6 months following stroke [110]. The authors used data from the EFFECTS trial to identify two post-stroke physical activity trajectories: those increasing their physical activity over time and those decreasing their physical activity over time. Increasing physical activity over time was associated with better functional outcomes. The same study found that participants without post-stroke cognitive impairment were more likely to be physically active, underlining the interconnection of cognition, physical activity, and disability in survivors of stroke. Although cognition can impact physical activity level after stroke, there is also evidence that physical activity following ischemic stroke may improve cognition and lower the risk of depression [111,112,113]. Future studies on the role of physical activity following stroke should aim to evaluate the type, timing, and dose of exercise that can be applied to different phenotypes of stroke survivors, given the inherent challenges in studying an intervention that likely does not apply in a one-size-fits-all approach.

#### 5.3.2. Diet and Nutrition

Both specific dietary changes following stroke as well as general diets for optimal brain health are actively being investigated. A 2021 open-label clustered randomized trial found that patients with a history of stroke or hypertension had reduced rates of an additional stroke when using salt substitutes over traditional salt [114]. These findings are in line with the AHA/ASA’s recommendation to limit sodium intake to 2300 mg and ideally <1500 mg per day, particularly in those with hypertension [53]. The Mediterranean-DASH Intervention for Neurodegenerative Delay (MIND) diet—similar to the Mediterranean or Dietary Approaches to Stop Hypertension (DASH) diet but with the addition of emphasizing consumption of leafy greens and berries—has mixed evidence with respect to preventing dementia and improving mood [115,116,117]. This choice of diet is now being studied in stroke survivors, with early evidence suggesting it may slow post-stroke cognitive decline, though more research is needed [118]. Diets that are composed of processed, high-fat, fried, and simple-carbohydrate foods are associated with greater post-stroke depressive symptoms, suggesting that the MIND diet may also improve post-stroke mood symptoms and lower the risk of post-stroke depression [119].

Additionally, malnutrition acutely following stroke may affect anywhere from 6% to 79% of survivors of stroke depending on the timing and method of ascertainment, and is associated with worse functional and recovery outcomes [120]. One large driver of malnutrition is post-stroke dysphagia, with current Canadian guidelines recommending using validated procedures to screen for dysphagia, pre-morbid malnutrition, and early consultation of speech language pathologists and dieticians [28].

#### 5.3.3. Smoking

Smoking cessation is currently recommended for secondary stroke prevention, but there is a large variability in initiating smoking cessation medications in hospitalized survivors of stroke as well as in quit rates among survivors of stroke [35,121,122]. A large observational study of stroke survivors found that never-smokers had the lowest rates of post-stroke dementia compared to patients who quit, sustained, or initiated smoking following stroke, with the most detrimental cognitive effects of smoking following stroke seen in younger patients [123]. Sustained smoking following stroke has also been associated with worse functional outcomes, in a dose-dependent manner [124]. Given the evidence tying smoking to various aspects of post-stroke brain health, smoking cessation is one of the most important modifiable factors to optimize brain health in stroke survivors. There are cross-sectional associations between depressive symptoms and persistence of smoking among stroke survivors, suggesting that smoking cessation and mood symptoms may be intertwined [125]. As such, clinicians targeting smoking habits should screen for and aggressively treat co-occurring mood disorders and related symptoms.

#### 5.3.4. Alcohol, Marijuana, and Substance Use Disorder

Unlike smoking tobacco, the role of alcohol in post-stroke brain health is more nuanced. Excessive alcohol intake is associated with incident stroke in all ages, though the evidence is mixed on the association of light alcohol consumption and stroke risk, since some studies suggest a potential protective effect [126,127,128,129,130]. One single-center study found a strong association between excessive alcohol intake and 30-day readmission among stroke survivors in a safety-net hospital [131]. Light alcohol consumption did not portend a strong association with either improved or worse functional outcomes in men in one study, though the referent group was individuals without strokes [128]. There is even less evidence on how tetrahydrocannabinol (THC) and/or cannabidiol (CBD) impact post-stroke brain health. One pilot study aimed to evaluate if the combination of THC and CBD would improve post-stroke spasticity, though it is unclear if the study was ever completed [132]. Substance use disorder, broadly defined, has been associated with increased risk of post-stroke mortality in one study [133]. However, to our knowledge, no dedicated research has evaluated the efficacy of different post-stroke substance use disorder interventions on improving post-stroke outcomes.

#### 5.3.5. Sleep

It is estimated that up to 50% of stroke survivors will have suboptimal sleep quantity and/or quality, with evidence suggesting that post-stroke sleep disorders are associated with worse cognitive, mood, and functional outcomes [134,135,136]. Nonetheless, only about 6% of stroke survivors undergo formal sleep evaluations [137]. However, the RCTs to date on the utility of continuous positive airway pressure (CPAP) improving cognition, mood, and recovery following stroke have mixed findings, with differences potentially due to limited study power as well as heterogeneity in treatment adherence rates, intervention timing, and targeting the correct patient populations [138]. For example, a meta-analysis of prior randomized clinical trials found that initiation of CPAP within 2 weeks of stroke in patients with obstructive sleep apnea improved global cognition, though initiation after 2 weeks of stroke was not beneficial [139]. The ongoing Sleep-SMART trial will be informative in delineating which, if any, group of stroke survivors is most likely to gain secondary prevention benefits from treating sleep apnea (URL: https://www.clinicaltrials.gov (accessed on 10 August 2023), unique identifier: NCT03812653). The current AHA/ASA guidelines on secondary stroke prevention recommend that “In patients with an ischemic stroke or TIA, an evaluation for OSA may be considered for diagnosing sleep apnea” as well as “In patients with an ischemic stroke or TIA and OSA, treatment with positive airway pressure (e.g., continuous positive airway pressure [CPAP]) can be beneficial for improved sleep apnea, blood pressure, sleepiness, and other apnea-related outcomes” [35]. Given the possible multi-modal benefits of treating sleep disorders following stroke for brain health and low risk of harm, we recommend a low threshold for testing for and treating sleep disorders in survivors of stroke, especially in those with post-stroke cognitive impairment or mood symptoms. Home sleep apnea testing can effectively identify sleep-disordered breathing, and effective in-clinic screening can take less than 5 min, making post-stroke sleep disorders a potentially modifiable determinant of post-ischemic stroke brain health [140,141]. However, more research is needed to precisely determine which stroke survivors are most likely to benefit from treating sleep disorders, when intervening is most beneficial, which specific domains of post-ischemic brain health are likely to benefit, and how newer non-CPAP treatment options impact outcomes.

**Table 2:** Recommended clinical considerations and areas for future research in lifestyle determinants of post-stroke brain health.

Target Area	Clinical Considerations	Level of Evidence	Areas for Future Research
*Physical activity*	Cardiopulmonary aerobic exercise can reduce multiple aspects of post-stroke disability and should be considered in all survivors of stroke [109].	Cochrane review, expert recommendations, post hoc analysis of clinical trial	Determining type, timing, and dose of physical activity is most effective in mitigating post-stroke physical and cognitive disability and how this varies by specific stroke and patient characteristics.
	Structured aerobic exercise should be conducted at least 3 days per week, lasting at least 20 min, and for at least 8 weeks in duration following stroke. However, there may be a dose–response effect; thus, any aerobic exercise is better than none, and more than the minimum recommendation may lead to additional benefits [110,142].		
	Physical activity is one of the most important factors for secondary prevention in patients with stroke from intracranial atherosclerosis and likely also follows a dose-dependent relationship. Increased walking may be a sustainable recommendation to help with secondary prevention in the setting of intracranial atherosclerosis [108].		
*Diet*	Counsel on lowering daily sodium intake in survivors of stroke to a goal of <1500–2300 mg, particularly in patients with comorbid hypertension [53,114].	Open-label cluster randomized clinical trial, guideline recommendations, observational research	Determine if certain diets can improve post-stroke cognitive trajectories and if this varies by stroke or patient characteristics.
	Encourage survivors of stroke to minimize processed foods and consider following the MIND diet [115,116,118,119].		
*Smoking cessation*	Consider pharmacological management to assist with smoking cessation following stroke paired with behavioral interventions [35,121].	Observational research, secondary prevention guidelines	Determine psychosocial, systemic, and other barriers to smoking cessation among survivors of stroke to more effectively intervene to improve smoking cessation rates following stroke.
	Consider screening and treating comorbid mood disorders in survivors of stroke who smoke [125].		
*Alcohol, Marijuana, and other substance use*	In individuals meeting criteria for either alcohol use disorder or excessive alcohol use (8+ alcoholic drinks/week for women, 15+ alcoholic drinks/week for men, or engaging in binge drinking), we recommend referral to addiction services following stroke [130].	Observational research	More research is needed to determine how light alcohol use impacts post-stroke functional and cognitive outcomes as well as recurrent stroke risk.
	For individuals with any substance use disorder, we recommend referral to addiction services following stroke [132,142,143,144].		More research is needed to determine how light THC and/or CBD use impacts post-stroke functional and cognitive outcomes.
	Coordination of care with addiction support services with tailored medication and/or behavioral plan should be planned before patients with comorbid stroke and substance use disorder leave the inpatient setting, particularly those with strokes from drug-related endocarditis [142,145].		More research is needed on how to improve post-stroke substance use disorder systems of care.
*Sleep*	Screen for and consider home sleep testing in patients with post-stroke cognitive decline or mood disorders; testing within 2 weeks following stroke may be the most beneficial time for testing and starting treatment, though more research is needed [134,139,140,141,143].	Observational research, randomized clinical trial, meta-analysis of clinical trials, expert opinion	Determine whether treating sleep-disordered breathing reduces stroke recurrence in patients that wear CPAP or other mask-based interventions >4 h per night.
	Consider screening for and testing for sleep apnea in patients with comorbid hypertension following stroke.		Determine real-world compliance rates with CPAP in patients with comorbid sleep apnea and history of stroke.
			Determine the efficacy of newer non-CPAP treatments such as hypoglossal nerve stimulation therapy in improving post-stroke cognition, mood, and mitigating stroke recurrence.
			Determine if certain patients with specific stroke or other characteristics are more likely to benefit from treatment of sleep-disordered breathing for mood, cognition, and functional recovery.

### 5.4. Social and Psychological Factors

Underpinning survivors’ ability to engage in post-stroke lifestyle modifications and adherence to medication management is psychosocial health. Changes in post-stroke mood, psychological well-being, and emotional state are likely complex and multifactorial, reflecting both direct effects of damage to networks involved in emotional regulation as well as related to large adjustments in identity and autonomy. Survivors of stroke may have changes in executive function, self-agency, and personality. Further, the roles of survivors within their families and communities may change drastically, likely contributing to post-stroke psychological trajectories and in turn brain health more broadly [144]. One study found the prevalence of post-stroke mood disorders reaching 5%, 16%, and 21% at 5 days, 1 month, and 3 months following stroke, respectively [145]. The study found that social support, disability level, and change in impairment following stroke were most strongly associated with mood disorders at 1 month following stroke, highlighting the interconnectedness of post-stroke mental health, brain health, and physical functioning. Table 3 displays clinical considerations and areas for future research with respect to post-stroke social and psychological factors. 

#### 5.4.1. Depression

Post-stroke depression is estimated to affect about one in three survivors of stroke and has been associated with worse functional, cognitive, and mortality outcomes [146,147,148]. Given its high prevalence and association with multiple components of brain health, the prevention and treatment of post-stroke depression remain a priority for clinicians and researchers alike. Since survivors of stroke may also have their language, affect, cognition, and emotional congruency affected, post-stroke depression can be difficult to diagnose. The Center of Epidemiological Studies-Depression Scale (CES-D), 21-item Hamilton Depression Rating Scale (HDRS), and the nine-item Patient Health Questionnaire (PHQ-9) all have a high degree of sensitivity and specificity for diagnosing post-stroke depression [146]. While some evidence suggests screening for depression may lead to better treatment and less depressive symptoms in stroke survivors, more research is needed to determine the optimal timing and delivery of depression screening and subsequent treatment [146]. Numerous studies suggest that pharmacological treatment and/or psychological therapy can reduce depressive symptoms in patients with post-stroke depression [149]. At this time, most guidelines and experts currently recommend pharmacological and/or psychological therapy to reduce the symptoms of post-stroke depression, with neuromodulation—the alteration or reorganization of synaptic connectivity or functional activity of the brain—also likely effective [28,146]. However, more research is needed on pairing different post-stroke depression etiologies and phenotypes with the most effective treatment. From a prevention perspective, analyses of two recent large, randomized trials had conflicting results on the efficacy of 20 mg of Fluoxetine decreasing incident post-stroke depression [150,151]. Pre-stroke depression is also relatively common and associated with worse post-stroke discharge destination, suggesting the possibility of depression impacting stroke survivors’ ability to meet the necessary therapy requirements for inpatient rehabilitation and should be considered during discharge planning [152].

#### 5.4.2. Other Post-Stroke Mood Sequelae

Aside from depression, there is a wide breadth of psychological and psychiatric sequelae of stroke, including but not limited to apathy, fatigue, pseudobulbar affect, and anxiety, all of which can adversely impact cognitive and functional recovery following stroke [28,153].

For instance, post-stroke fatigue seems to be highly prevalent, with one study reporting estimates at 50% or more of survivors of stroke experiencing fatigue, including in survivors of minor strokes [154,155]. While still under investigation, some potential determinants of post-stroke fatigue include thalamic ischemia, the degree of pre-morbid cerebral white matter disease, stroke severity, and the presence of post-stroke depression [156]. Modafinil has been shown to be effective for reducing post-stroke fatigue in the Modafinil in Debilitating Fatigue After Stroke (MIDAS) trial [157].

Also common, about one in three survivors of stroke experience apathy, with current evidence suggesting it results from damage to neural networks rather than a specific structural location, though the anterior cingulate cortex (ACC) and nucleus accumbens may anchor these networks [158]. One small clinical trial found that escitalopram or problem-solving therapy may help to prevent post-stroke apathy, though there are no conclusive data to support pharmacologic treatment of post-stroke apathy [159].

Pseudobulbar affect—involuntary crying, laughing, or other physical or emotional responses incongruent with mood—is estimated to affect anywhere between 11 and 52% of survivors of stroke. It is theorized to result from damage to glutaminergic, serotoninergic, and dopaminergic neuronal circuits of the corticolimbic–subcortico–thalamic–pontocerebellar network [160]. The combination of Dextromethorphan and Quinidine has been shown to reduce lability and decrease episodes of post-stroke pseudobulbar affect in stroke survivors in a phase-two trial, and selective serotonin reuptake inhibitors (SSRIs) and other anti-depressants may also be beneficial [161,162].

#### 5.4.3. Social Interactions and Support

A recent study found that optimism following stroke was associated with improved recovery, highlighting the importance of the brain–mind–body axis independent of known psychiatric diagnoses [163]. Key to optimizing the brain–mind–body axis is ensuring stroke survivors maintain robust social interactions. Social support has been associated with improved recovery and cognitive resilience and reduced risk of mortality following stroke [164,165,166]. However, translations of these findings to effective interventions mitigating the detrimental effects of social isolation in stroke survivors have been limited. A trial evaluating the efficacy of eight visits of intensive support (motivational interviewing and feedback) over two years compared to usual care found improved rates of secondary prevention targets but not recurrent vascular events or death [167]. Additionally, there is qualitative evidence of peer support groups mitigating subjective social isolation and improving hopefulness among individuals with acquired brain pathologies such as stroke, though quantitative research is needed to determine the impact of such interventions on psychological, cognitive, physical, and secondary prevention outcomes [168,169]. During the COVID-19 pandemic, a wide range of remote interventions to mitigate social isolation in the elderly were developed [170]. Reconfiguring and studying these interventions for stroke survivors experiencing social isolation is one possible future avenue to mitigate the harmful effects of social isolation.

Additionally, an estimated 25–54% of family members of survivors of stroke experience caregiver burden: the additional physical, psychosocial, emotional, or financial strain borne by informally caring for a survivor of stroke [171]. Caregiver burden has been associated with functional disability among survivors of stroke, suggesting a potential bidirectional relationship between stroke recovery and the health of caregivers of stroke [172,173]. Interventions to reduce caregiver burden include education on taking on the caregiver role, financial assistance, mental health counselling, respite care, and referral to community services [174].

**Table 3:** Recommended clinical recommendations and areas for future research in psychosocial determinants of post-stroke brain health.

Target Area	Clinical Considerations	Level of Evidence	Areas for Future Research
*Depression*	Consider whether pre-stroke depression is a modifiable factor affecting stroke survivors’ ability to meet the therapy requirements needed for inpatient rehabilitation.	Observational research, guideline recommendations, Cochrane review, expert scientific statement	Determine if and when the optimal time to screen for post-stroke depression is.
	Screen for depression with the PHQ-9, CES-D, and HDRS in survivors of stroke if not meeting anticipated functional goals or experiencing cognitive decline [28,149].		
	Recognize that aphasia can make diagnosing depression or another comorbid mood disorder difficult and may warrant discussing with caregivers and family.		Determine which patients, if any, may benefit from interventions aimed at preventing post-stroke depression.
	Treat post-stroke depression with medication classes such as SSRIs, SNRIs, or Mirtazapine based off phenotype and comorbidities; consider referral for psychological therapy; and consider referral for neuromodulation treatments [146,149].		Develop treatments tailored to different phenotypes and etiologies (i.e., from infarct eloquent mood networks vs. from post-stroke physical disability)
*Other Post-Stroke Mood Sequelae*	Consider screening for anxiety, fatigue, and other modifiable mood sequelae, particularly in those with unexpected cognitive/functional decline or plateau.	Phase II clinical trials, Cochrane review	Determine if and when the optimal time to screen for non-depression mood sequelae of stroke is.
	Consider modafinil for the treatment of post-stroke fatigue if no underlying treatable condition found (i.e., hypothyroidism or sleep apnea) [160].		
	Consider Dextromethorphan/Quinidine or SSRIs for the treatment of pseudobulbar affect [161,162].		
*Social Isolation*	Consider screening for social isolation, particularly in elderly stroke survivors or those with other mood disorders [146,175,176].	Observational research, expert scientific statement, systematic review	Determine the role of peer-support groups in improving psychological well-being, functional status, and cognition following stroke.
			Determine the efficacy of remote and telehealth interventions in mitigating social isolation following stroke.
	Consider assessing caregiver well-being in caregivers of stroke survivors, particularly survivors of stroke with high levels of disability [171,177].		

## 6. Future Directions

In this review, we have provided an overview of multiple clinically relevant domains of brain health in the context of stroke. One ongoing area of clinical–translational research is utilizing wearable sensors, mobile-phone-based applications, telehealth interventions, and other wireless technology to track and monitor the cognitive and physical well-being of stroke survivors. The real-time information and feedback on motor function afforded by these innovations may improve physical activity and motor recovery, as well as improve access to recovery interventions [178,179,180]. Cramer et al. demonstrated that telerehabilitation for upper extremity recovery over 36 sessions for individuals who experienced a stroke 4 to 36 weeks prior to enrollment is non-inferior to in-person rehabilitation as measured by the Fugl-Meyer Arm Motor Scale [181]. A subsequent study by Cramer et al. demonstrated that a home-based telehealth system reliably sends automatic actionable healthcare reports to clinicians for the care of patients with stroke [182]. One pilot feasibility study showed that in-home virtual reality in the chronic phase of stroke has the potential to improve activities of daily living and reduce pain [183]. Another feasibility study showed that a therapist-guided, tablet-based application for post-stroke aphasia is both adaptable to individual needs and viable from both patients’ and therapists’ perspectives [184]. As telerehabilitation in all physical, cognitive, and psychosocial domains of post-stroke brain health continues to improve, we are optimistic that remote monitoring and interventions for stroke survivors will lead to more personalized and widely accessible care [185,186,187]. Future work in telerehabilitation should continue to focus on specific actionable outcome measures, as well as move from feasibility and efficacy studies towards implementation and scalability studies in communities where post-stroke rehabilitation care is hardest to obtain.

Neuromodulation, already mentioned earlier in the context of post-stroke depression, is a growing field aimed at optimizing many facets of post-stroke brain health. Vagal nerve stimulation can improve upper extremity function when initiated nine months or longer from the time of stroke and is approved by the FDA for this use [188]. Different forms of transcranial magnetic stimulation (TMS) are being studied, to advance our understanding of stroke recovery mechanisms and as a therapy to enhance neuroplasticity and recovery [189,190]. Both TMS and transcranial direct current stimulation (tDCS) show promise for improving language recovery and treating post-stroke depression [191,192,193]. We expect the field of post-stroke neuromodulation to become more precise and targeted with respect to the brain area targeted; treatment timing, duration, and dosage; pairing with other treatments; and identifying the target patient population.

The role of stroke survivors’ surrounding environment is increasingly being studied as a determinant of post-stroke brain health. The degree of environmental enrichment—how an environment increases multi-modal sensory stimulation and promotes increased physical and cognitive recovery—may help facilitate post-stroke neuroplasticity [194]. In animal models, an enriched environment improved post-stroke functional and cognitive recovery. Enriched environments in rat models of middle cerebral artery occlusion strokes and transient ischemic attacks have demonstrated multiple potential mechanisms of benefit: proangiogenic reorganization, progenitor cell proliferation, and neuronal differentiation in the penumbra, neuronal autophagy in the penumbra, and decreased markers of neuroinflammation and oxidative stress [195,196,197,198]. Rats with enriched environments performed better on post-stroke Morris water maze tests and had lower modified neurological severity scores [195,196,197,198]. However, the largest clinical trial to date in human stroke survivors did not find a beneficial effect of enriched environments [199]. More research is needed to most accurately translate findings from animal to human studies and to match specific enriched environments to appropriate patient populations, in order to maximize the benefit of this potential intervention.

While much research has been dedicated to improving specific vascular–metabolic comorbidities, there is a need to address vascular and metabolic health more comprehensively in patients with stroke, as many vascular–metabolic conditions co-occur and share similar modifiable risk factors. Few high-fidelity clinical trials have evaluated whether multi-domain intensive vascular risk factor control can improve post-stroke brain health. The Austrian Polyintervention Study to Prevent Cognitive Decline after Ischemic Stroke (ASPIS) study found that intensive management of clinical therapy, blood pressure control, lipid and glycemic control, healthy diet, regular physical activity, and cognitive training did not lead to better post-stroke cognition as measured by the ADAS-Cog score or worsening of two of five cognitive domains 24 months following stroke compared to standard care [200]. However, the authors noted the limitations of a small sample size, recruitment of patients with predominantly mild strokes, and a short follow-up period. More studies looking at multi-domain vascular risk factor control on the impact of post-stroke cognition are needed, with carefully selected inclusion criteria with respect to stroke severity and location, patient age, and follow-up duration. Pragmatic trials and real-world studies may provide useful insights in this context given the cost and recruitment difficulties of standard clinical trials with multi-modal interventions [201].

The concept of the neurovasculome has emerged recently, with evidence suggesting that the interplay between brain cells, meninges, blood vasculature, and lymphatic vasculature is a major determinant of cognition in individuals with and without an ischemic stroke [202]. The role of this vascular–cerebral interface is increasingly being studied and may represent a pharmacological target for mitigation of post-stroke cognitive decline. A recent trial used isosorbide mononitrate and cilostazol to target endothelial dysfunction and found that the combination of these two agents improved cognition and functional status one year after a lacunar infarct, supporting the neurovasculome theory [103].

In addition to an individualized approach to post-stroke brain health, mitigating known factors that lead to population disparities in post-stroke brain health needs to be prioritized. Many cognitive and physical outcomes after stroke are worse among racial minorities, patients with lower socioeconomic status, and those living in disadvantaged neighborhoods, suggesting structural determinants are at play in shaping post-stroke brain health [203,204,205,206]. For example, stroke survivors experiencing housing insecurity tend to have worse acute and post-stroke outcomes [207]. As stroke is a socially and racially patterned disease, future work should address known racial and socioeconomic inequities in post-stroke brain health [208]. Potential means for decreasing inequities in post-stroke brain health include ensuring representation of diverse populations in future clinical trials and research, using measures of disparity reduction as endpoints in clinical research, improving access to optimal post-stroke care, and reducing barriers to translation of research findings to historically marginalized communities.

## 7. Conclusions

In this review, we have provided a broad overview of the field, provided specific actionable recommendations for integrating monitoring and management of post-stroke brain health into clinical practice, and identified knowledge gaps of the field ripe for research. We describe potential future interventions in the field on the horizon. As one of the first reviews on the topic, we hope that we have spurred the need for more clinical and research attention on this important aspect of stroke care.

In closing, major developments have occurred in acute stroke care over the past decade. The indications for acute interventions continue to expand, refinement of secondary prevention regimens is regularly informed by new evidence, and methods for identifying specific stroke etiologies in patients that would previously have been defined as cryptogenic are continuously improving. This recent progress is truly remarkable, and the same vigor, excitement, creativity, and innovation are needed for evaluating and improving brain health after stroke.

## Data Availability

No new data was created and no pre-existing dataset was analyzed given this is a narrative review manuscript. BioRender content included in the completed graphic (Figure 1) is not licensed for any commercial uses beyond publication in a journal.
